# Familial Mesial Temporal Lobe Epilepsy: Clinical Spectrum and Genetic Evidence for a Polygenic Architecture

**DOI:** 10.1002/ana.26765

**Published:** 2023-08-31

**Authors:** Rebekah V. Harris, Karen L. Oliver, Piero Perucca, Pasquale Striano, Angelo Labate, Antonella Riva, Bronwyn E. Grinton, Joshua Reid, Jessica Hutton, Marian Todaro, Terence J. O'Brien, Patrick Kwan, Lynette G. Sadleir, Saul A. Mullen, Emanuela Dazzo, Douglas E. Crompton, Ingrid E. Scheffer, Melanie Bahlo, Carlo Nobile, Antonio Gambardella, Samuel F. Berkovic

**Affiliations:** ^1^ Epilepsy Research Centre, Department of Medicine (Austin Health) The University of Melbourne Heidelberg Victoria Australia; ^2^ Population Health and Immunity Division Walter and Eliza Hall Institute of Medical Research Parkville Victoria Australia; ^3^ Department of Medical Biology The University of Melbourne Parkville Victoria Australia; ^4^ Bladin‐Berkovic Comprehensive Epilepsy Program, Department of Neurology Austin Health Heidelberg Victoria Australia; ^5^ Departments of Medicine and Neurology, Royal Melbourne Hospital The University of Melbourne Melbourne Victoria Australia; ^6^ Department of Neurology Alfred Health Melbourne Victoria Australia; ^7^ Department of Neuroscience, Central Clinical School Monash University Melbourne Victoria Australia; ^8^ IRCCS Istituto Giannina Gaslini, Member of ERN‐Epicare Genoa Italy; ^9^ Departments of Neurosciences, Rehabilitation, Ophthalmology, Genetics, Maternal, and Child Health University of Genoa Genoa Italy; ^10^ Neurophysiopatology and Movement Disorders Clinic University of Messina Messina Italy; ^11^ Institute of Neurology, Department of Medical and Surgical Sciences Magna Graecia University of Catanzaro Catanzaro Italy; ^12^ Department of Paediatrics and Child Health University of Otago Wellington New Zealand; ^13^ The CNR Institute of Neuroscience (CNR‐IN), National Research Council of Italy Padova Italy; ^14^ Department of Neurology Northern Health Epping Victoria Australia; ^15^ Florey Institute of Neuroscience and Mental Health Melbourne Victoria Australia; ^16^ Murdoch Children's Research Institute and Department of Paediatrics University of Melbourne, Royal Children's Hospital Melbourne Victoria Australia

## Abstract

**Objective:**

Familial mesial temporal lobe epilepsy (FMTLE) is an important focal epilepsy syndrome; its molecular genetic basis is unknown. Clinical descriptions of FMTLE vary between a mild syndrome with prominent déjà vu to a more severe phenotype with febrile seizures and hippocampal sclerosis. We aimed to refine the phenotype of FMTLE by analyzing a large cohort of patients and asked whether common risk variants for focal epilepsy and/or febrile seizures, measured by polygenic risk scores (PRS), are enriched in individuals with FMTLE.

**Methods:**

We studied 134 families with ≥ 2 first or second‐degree relatives with temporal lobe epilepsy, with clear mesial ictal semiology required in at least one individual. PRS were calculated for 227 FMTLE cases, 124 unaffected relatives, and 16,077 population controls.

**Results:**

The age of patients with FMTLE onset ranged from 2.5 to 70 years (median = 18, interquartile range = 13–28 years). The most common focal seizure symptom was déjà vu (62% of cases), followed by epigastric rising sensation (34%), and fear or anxiety (22%). The clinical spectrum included rare cases with drug‐resistance and/or hippocampal sclerosis. FMTLE cases had a higher mean focal epilepsy PRS than population controls (odds ratio = 1.24, 95% confidence interval = 1.06, 1.46, *p* = 0.007); in contrast, no enrichment for the febrile seizure PRS was observed.

**Interpretation:**

FMTLE is a generally mild drug‐responsive syndrome with déjà vu being the commonest symptom. In contrast to dominant monogenic focal epilepsy syndromes, our molecular data support a polygenic basis for FMTLE. Furthermore, the PRS data suggest that sub‐genome‐wide significant focal epilepsy genome‐wide association study single nucleotide polymorphisms are important risk variants for FMTLE. ANN NEUROL 2023;94:825–835

Temporal lobe epilepsy (TLE) is the most common focal epilepsy in adulthood.^1^ Initially regarded as an acquired disorder, two forms of familial TLE are now widely recognized.^2^ First, a lateral form, autosomal dominant epilepsy with auditory features (ADEAF),^3^ associated with pathogenic variants in *LGI1* is in about half the cases.^4^ Second, the more common mesial form, familial mesial temporal lobe epilepsy (FMTLE).^5^, ^6^, ^7^, ^8^ FMTLE was first described in twins and small families as a benign syndrome with adolescent or adult seizure onset and no preceding febrile seizures (FS).^5^ Electroencephalogram (EEG) was often uninformative and magnetic resonance imaging (MRI) of the brain appeared normal. Affected individuals had a favorable prognosis. Pedigree segregation analyses implicated a polygenic etiology.^7^ Consistent with this, monogenic gene discovery has been inconclusive except for some small dominant families with pathogenic variants in the GATOR1 complex genes *(DEPDC5*, *NPRL2*, and *NPRL3*).^9^, ^10^, ^11^

Whereas hippocampal sclerosis (HS) and FS were initially not thought to be characteristic of FMTLE, some families have since been described with these features.^12^, ^13^, ^14^, ^15^ Outcomes in these families vary, including drug‐resistant epilepsy. Preceding FS have been reported in FMTLE more frequently than in the general population,^14^, ^16^ but less than typically seen in sporadic mesial TLE (MTLE) with HS (where they have been reported in over 70% of cases in one study).^17^ Families have also been described with individuals with simple FS and others with MTLE occurring in independent family members with a small proportion having both.^15^, ^18^ This suggests a possible shared genetic basis of FS and MTLE. The genetic architecture in these families is largely unknown, although rare dominant families have been reported with causative mutations in the sodium channel gene *SCN1B*.^18^ In addition, there is emerging evidence for shared common risk variants in FS and focal epilepsy. The recent International League Against Epilepsy (ILAE) genomewide association study (GWAS) found a significant genetic correlation between focal epilepsy and FS.^19^

Familial focal epilepsy is genetically heterogeneous; some monogenic forms are described^20^ with FMTLE being largely unsolved (see above). Although GWAS results across all focal epilepsies show some single‐nucleotide polymorphism (SNP)‐based heritability, no consistent genomewide significant loci have been identified in the ILAE GWAS despite including > 16,000 focal epilepsy cases and 50,000 controls.^19^, ^21^

Using GWAS data to calculate polygenic risk scores (PRS)–a weighted sum of the number of common epilepsy risk alleles an individual carries–has proven less powerful for focal epilepsy compared to genetic generalized epilepsy.^22^, ^23^ Furthermore, in a cohort of individuals with focal epilepsy and a family history of epilepsy, no significant enrichment of PRS for focal epilepsy (generated using data from the 2018 “ILAE 2” GWAS) was observed compared to population controls.^22^ Given the heterogeneous nature of the focal epilepsies, genetic analysis of a more homogeneous focal epilepsy subcohort, such as FMTLE, may be more informative.

This study aimed to (1) describe the clinical features of our large FMTLE cohort and (2) test whether common risk variants associated with focal epilepsy and/or FS, as measured by PRS, are enriched in FMTLE families.

## Methods

### Cohorts

We established a multicenter international collaboration to identify FMTLE families. Families were selected based on identifying individuals with MTLE and a family history of seizures. About two‐thirds of potentially eligible families, comprising affected as well as unaffected individuals, were studied at the Epilepsy Research Centre, Melbourne, Australia, as part of a long‐standing study into the genetic basis of epilepsy. The remaining families were referred by neurologists at Alfred Health and The Royal Melbourne Hospital (both in Melbourne, Australia), the University of Otago (Wellington, New Zealand), University of Genoa (Genoa, Italy), and Magna Graecia University (Catanzaro, Italy).

### Ethics Statement

All participants, or their legal guardians, provided signed informed consent according to local institutional review board requirements. This study was approved by the Austin Health Human Research Ethics Committee (H2007/02961) and the Walter and Eliza Hall Institute of Medical Research Ethics Committee (G20/01). The QSkin Sun and Health Study was approved by the QIMR Berghofer Human Research Ethics Committee (P1309 and amended for use in this study: P2034).

### Classification of Individuals

We devised criteria to classify affected individuals into definite, probable, or possible MTLE according to diagnostic certainty for MTLE based on a combination of electro‐clinical evidence (Table 1). Support for an MTLE diagnosis was categorized as (1) strong, (2) moderate, or (3) low. “Strong” support for an MTLE diagnosis included EEG evidence of a seizure starting over the temporal lobe, hippocampal sclerosis on MRI, or classic mesial temporal seizure phenomena^7^: déjà vu, stereotyped flashbacks of a past event, a rising epigastric/visceral sensation, or stereotyped (and usually noxious) olfactory or gustatory hallucinations. “Moderate” support for an MTLE diagnosis included interictal temporal epileptiform activity on EEG, or clinical features suggestive of an MTLE diagnosis but which on their own were not sufficiently localizing. These features included: dreamlike sensation, fear, nausea, warmth, sweating, flushing, or pallor. Low support included temporal slowing on EEG, or nonspecific focal clinical features, such as unilateral motor activity, and head or eye version to one side. Individuals with “suspicious déjà vu,” defined as intense, prolonged, and frequent déjà vu, that could occur in clusters but did not progress to loss of awareness or a bilateral tonic–clonic seizure were included as “possible MTLE”^8^ (see Table 1).

**Table 1:** Criteria for MTLE Diagnostic Certainty

Individual diagnostic certainty	Level of support
Definite MTLE	2 strong support OR
	1 strong AND 1 moderate support of different category^a^
Probable MTLE	1 from strong support alone OR
	2 from moderate support
Possible MTLE	1 from moderate support AND
	1 from low support of different category^a^ OR
	suspicious déjà vu

*Note*: Strong support included classic MTLE seizure phenomena, or a seizure starting over the temporal lobe captured on EEG, or HS on MRI. Moderate support included seizure phenomena suggestive of MTLE but which on their own are not sufficiently localizing, or temporal epileptiform interictal activity on EEG. Low support included non‐specific focal seizure phenomena (eg, ictal head version) or temporal slowing on EEG.

^a^ There were 3 categories of diagnostic evidence: clinical seizure phenomena, EEG evidence, and MRI evidence.

Abbreviations: EEG = electroencephalogram; HS = hippocampal sclerosis; MRI = magnetic resonance imaging; MTLE = mesial temporal lobe epilepsy.

In addition, individuals with unclassified focal epilepsy were noted. They were defined as anyone with focal epilepsy which could not be localized, according to ILAE guidelines^24^, ^25^ and were not included in the FMTLE cohort. We also excluded individuals with epilepsy with auditory features (EAFs), acquired (lesional) epilepsies, MRI‐evidence of epileptogenic abnormalities other than HS, or a pathogenic variant in a known monogenic epilepsy gene (eg, *DEPDC5*, *NPRL2*, etc.); systematic whole exome sequencing was not done but half the families had a least one subject screened and no significant findings emerged.

### Classification of Families

Families were considered to be FMTLE when 2 or more first or second‐degree relatives were individually classified as having MTLE. Furthermore, at least one of the family members with MTLE was required to meet our definite or probable criteria (ie, a clear history of mesial ictal semiology). We excluded families with genetic epilepsy with febrile seizures plus (GEFS+).^26^ We also limited our study to families of European ancestry due to the planned genetic analyses and comparisons being restricted to European‐derived GWAS data.

### Comparison of Published and Unpublished Families

Clinical features from our published and unpublished cohorts were compared using 2‐tailed chi‐square test or 2‐tailed unpaired *t* test, as appropriate. Bonferroni adjustments were applied for multiple comparisons.

### Genetic Analysis

We genotyped all available individuals from FMTLE families with definite, probable, or possible MTLE. Unaffected relatives with DNA available were also genotyped, provided they were first‐degree relatives of an affected individual. However, we then excluded any families with members included in either the focal epilepsy or FS GWAS from which our planned PRS models were derived.^21^, ^27^ Population controls were obtained from the QSkin Sun and Health Study, a prospective cohort study of men and women, randomly sampled from the Australian state of Queensland.^28^

FMTLE family members and population controls were genotyped using the Illumina Global Screening Array (San Diego, CA, USA) at Erasmus MC in Rotterdam, The Netherlands. Genotyping was performed in 3 batches; 2 in 2019 using array version GSAMD‐v1 and one in 2022 using GSAMD‐v3. Each batch contained at least one duplicate sample as a technical control for the different genotyping batches.

We performed standard quality control (QC) measures using PLINK version 1.9.^29^ First, we removed all SNPs with discordant genotype calls for the technical control samples. We then excluded SNPs that exhibited high “missingness” rates (> 5%) or low minor allele frequency (< 35%). We also excluded samples with a high proportion of missing genotypes (> 10%) or those with sex mismatch between genetically inferred and reported genders. We generated pairwise identity‐by‐descent estimates with PLINK version 1.9 to confirm pedigree structures and to exclude the possibility of cryptic relatedness (proportion identity by descent > 0.0625) between any of the families or controls. Finally, principal component analysis for ancestry was performed on our final cohort, merged with 1,000 Genomes data, using PC‐Air to account for related samples.^30^ We did this to confirm that the population controls were a good ancestral match for the cases and to allow exclusion of samples that did not cluster with the European super population.

SNP imputation to the HRC r1.1 2016 (GRCh37/hg19) reference panel was performed using Minimac4 as implemented on the Michigan Imputation Server 23 with pre‐imputation phasing using Eagle version 2.4.^31^ Post‐imputation, we selected high‐quality imputed and genotyped SNPs (imputation quality scores *R* ^2^ > 0.9) and repeated the above standard QC measures.

Polygenic risk scores (PRS) were calculated using PRSice‐2^32^ for FMTLE family members and population controls using SNP effect sizes from the 2018 ILAE 2 focal epilepsy GWAS^21^ and a large Danish FS GWAS.^27^ As a negative control experiment, we also calculated PRS based on the 2018 ILAE 2 genetic generalized epilepsy (GGE) GWAS.^21^ PRSice‐2 follows the clumping and thresholding method for calculating PRS. First, SNPs are pruned to an uncorrelated subset (*r* ^2^ < 0.1 within 500 kb from the most significant SNP at each locus). The PRS is then calculated by summing over all SNPs meeting a *p* value threshold, with each SNP weighted by its effect size derived from the relevant summary statistic GWAS data.

We used a significance threshold of *p* < 0.1 for SNP inclusion in the PRS model for focal epilepsy (n = 25,903 SNPs), FS (n = 28,989 SNPs), and GGE (n = 18,599). We set our *p* value threshold to 0.1, as this has previously been determined as the optimal *p* value threshold for focal epilepsy PRS prediction.^23^ All PRS values were normalized to a standard normal distribution with a mean of zero and standard deviation of one. Statistical comparisons were made using linear mixed effects models using the lmekin function in R version 4.2.0. All regression models included sex and the first 5 genetic ancestry principal components as fixed‐effects covariates, and family identifiers as a random‐effects covariate.

## Results

### Melbourne Cohorts

**Figure 1:**
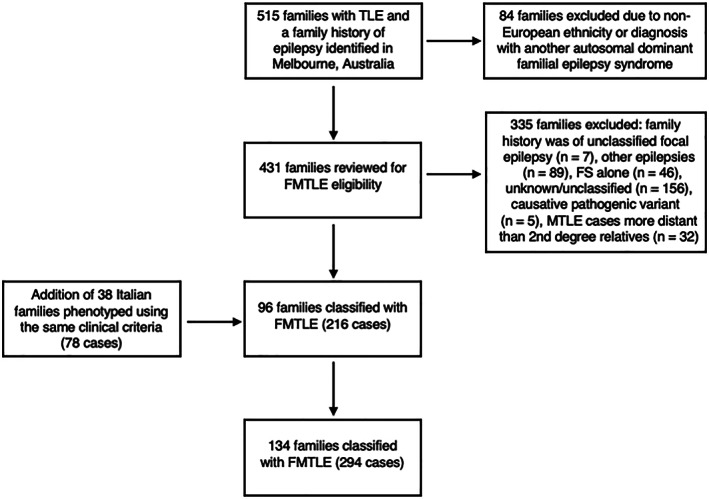
Process of review and inclusion of families to this study. FS = febrile seizure; FMTLE = familial mesial temporal lobe epilepsy; MTLE = mesial temporal lobe epilepsy; TLE = temporal lobe epilepsy.

Five hundred and fifteen families with at least one member with suspected TLE and a known family history of seizures were identified from the Epilepsy Programs at the Epilepsy Research Centre, Alfred Health, and The Royal Melbourne Hospital. Of these families, 84 were excluded due to known non‐European ethnicity or due to the family history being consistent with an autosomal dominant familial epilepsy syndrome (eg, GEFS+, ADEAF, autosomal dominant sleep‐related hypermotor epilepsy, self‐limited familial neonatal/infantile epilepsy, or familial adult myoclonic epilepsy). This left 431 families for review. Reasons for exclusion after review were: family history was of unclassified focal epilepsy (n = 7) or of other epilepsies (n = 89); family history of FS only (n = 46); family history was of an unknown/undefined epilepsy type (n = 156); family genetically solved with a pathogenic variant in *NPRL2*, *NPRL3*, or *SCN1B* (n = 5); or MTLE cases were more distant than second degree relatives (n = 32). After exclusions, 96 families (216 cases) were classified as having FMTLE and deemed eligible for clinical analysis. This process of review and inclusion to this study is illustrated in Figure 1.

### Italian Cohorts

Two further cohorts from Italy were also included in the analysis cohort. All families were evaluated for eligibility using the same diagnostic criteria as above. A total of 38 families with FMTLE (78 cases) were ascertained from the University of Genoa and Magna Graecia University. A summary of eligible families is shown in Table 2.

**Table 2:** Contribution of 3 Cohorts to Eligible Familial Mesial Temporal Lobe Epilepsy Families

	Melbourne (ERC, AH, and RMH)	University of Genoa	Magna Graecia University of Catanzaro	Total
MTLE cases	216	50	28	**294**
FMTLE families	96	16	22	**134**

Abbreviations: AH = Alfred Hospital; ERC = Epilepsy, Research Centre, Austin Health; FMTLE = familial mesial temporal lobe epilepsy; MTLE = mesial temporal lobe epilepsy; RMH = Royal Melbourne Hospital.

### Clinical Characteristics

Overall, 134 families were classified as having FMTLE. These comprised 294 recruited cases, of whom 173 (59%) were female. One hundred and one cases had definite MTLE, 147 probable MTLE, and 46 possible MTLE (including 13 with suspicious déjà vu). Of the 134 families included in this analysis, 58 have been reported earlier.^5^, ^7^, ^8^, ^16^, ^33^

Within these families, a further 226 individuals were reported as having a history of seizures (all no greater than second‐degree relatives from a case with MTLE). Of these individuals, we consider 45 likely to fit the above criteria for MTLE, based on family report, but were not directly studied. Additionally, 36 had unclassified focal epilepsy, 48 had FS alone, and 14 had well‐documented other epilepsy types (idiopathic generalized epilepsy [5], other focal epilepsies [7], and developmental and epileptic encephalopathies [2]). In 83 individuals, seizures were reported but no further detail was available. The number of individuals with MTLE per FMTLE family, including those not studied, ranged from 2 to 6 (mean = 2.5, median = 2, interquartile range [IQR] = 2–3).

The clinical characteristics of the 294 studied cases with definite, probable, or possible MTLE are summarized in Table 3. Déjà vu was the most common focal seizure symptom (183, 62%), followed by a rising epigastric sensation (101, 34%), a sense of fear, anxiety, or impending doom (64, 22%), a feeling of being in a dream, of time slowing down, or out of body experiences (52, 18%), and stereotyped olfactory or gustatory hallucinations (38, 13%). Twenty‐one cases (7%) reported auditory symptoms during their seizures, but these features followed the occurrence of mesial temporal phenomena. Nearly two‐thirds of cases (168, 57%) experienced one or more focal to bilateral tonic‐clonic seizure, which were infrequent. The exact age of onset of afebrile seizures was available for 249 cases and ranged from 2.5 to 70 years (mean = 21.6 years, median = 18 years, IQR = 13–28 years). Only a minority of cases had drug‐resistant epilepsy (34, 12%).

**Table 3:** Clinical Characteristics of 294 FMTLE Cases With Definite, Probable, or Possible MTLE

Features	Cases with MTLE, n (%)
Déjà vu	183 (62%)
Epigastric aura	101 (34%)
Noxious taste or smell	38 (13%)
Fear	64 (22%)
Nausea	54 (18%)
Dreamlike sensation	52 (18%)
Auditory features	21 (7%)
TCS	168 (57%)
HS^a^	27 (13%)
Drug‐resistant epilepsy	34 (12%)
Antecedent FS	48 (16%)
Range age of onset^b^	2.5–70 yr
Mean age of onset^b^	21.6 yr
Median age of onset (IQR)^b^	18 yr (13–28)

^a^ 215 cases had MRI results available.

^b^ 249 cases had exact age of onset data.

Abbreviations: FMTLE = familial mesial temporal lobe epilepsy; FS = febrile seizures; HS = hippocampal sclerosis; IQR = interquartile range; MRI = magnetic resonance imaging; MTLE = mesial temporal lobe epilepsy; TCS = tonic–clonic seizures.

A small subset (48, 16%) had a history of FS. Of these cases, nearly half (21/48, from 10 families) had at least one relative with MTLE and preceding FS. Of 215 cases with available MRI results, 27 (13%) had features of HS. Of these cases, nearly half (11/27, from 5 families) had at least one other family member with HS.

We compared our cases from 76 unpublished families to the 58 families we previously reported (Table 4). The only significant differences were that unpublished cases (n = 137) had a higher prevalence of drug‐resistant epilepsy (18% vs 5%, *p* = 0.0048, and HS (19% vs 6%, *p* = 0.026), and lower prevalence of déjà vu (53% vs 70%, *p* = 0.037) than previously reported cases (n = 157), with these differences likely reflecting the different ascertainment methods of our previous FMTLE studies.^7^, ^8^

**Table 4:** Comparison of Clinical Characteristics of Cases with MTLE Between Previously Published and Unpublished FMTLE Families

Feature	157 cases from 58 published families,^5^, ^7^, ^8^, ^16^, ^33^ n (%)	137 cases from 76 unpublished families, n (%)	*P*
Déjà vu	110 (70%)	73 (53%)	0.037
Epigastric aura	50 (32%)	51 (37%)	1
Noxious taste or smell	24 (15%)	14 (10%)	1
Fear	33 (21%)	31 (23%)	1
Nausea	28 (18%)	26 (19%)	1
Dreamlike sensation	25 (16%)	27 (20%)	1
Auditory features	16 (10%)	6 (4%)	0.71
TCS	86 (55%)	87 (64%)	1
HS^a^	6 (6%)	21 (19%)	0.026
Drug‐resistant epilepsy	8 (5%)	25 (18%)	0.0048
Antecedent FS	22 (14%)	26 (19%)	1
Range age of onset^b^	2.5–63 yr	2.5–70 yr	
Mean age of onset^b^	20.5 yr	22.8 yr	1
Median age of onset (IQR)^b^	17 yr (12–26)	18.5 yr (13–29)	

^a^ One hundred seven published and 108 unpublished cases had available MRI results.

^b^ One hundred thirty‐three published cases and 116 unpublished cases had exact age of onset data.

Abbreviations: FMTLE = familial mesial temporal lobe epilepsy; FS = febrile seizures; HS = hippocampal sclerosis; IQR = interquartile range; MTLE = mesial temporal lobe epilepsy; TCS = tonic–clonic seizures.

### Genetic Analysis

Of the 294 cases from 134 FMTLE families, 227 cases (123 families) were genotyped. Reasons for exclusion included no DNA sample available (n = 29) or QC failure (n = 28). Ten MTLE cases from 5 families were also excluded as a precautionary measure due to their inclusion in the 2018 ILAE 2 focal epilepsy GWAS from which the PRS were derived.^21^, ^27^

PRS were calculated for affected (n = 227) and unaffected individuals (n = 124) from FMTLE families, as well as population controls (n = 16,077).

### Polygenic Risk Scores

**Figure 2:**
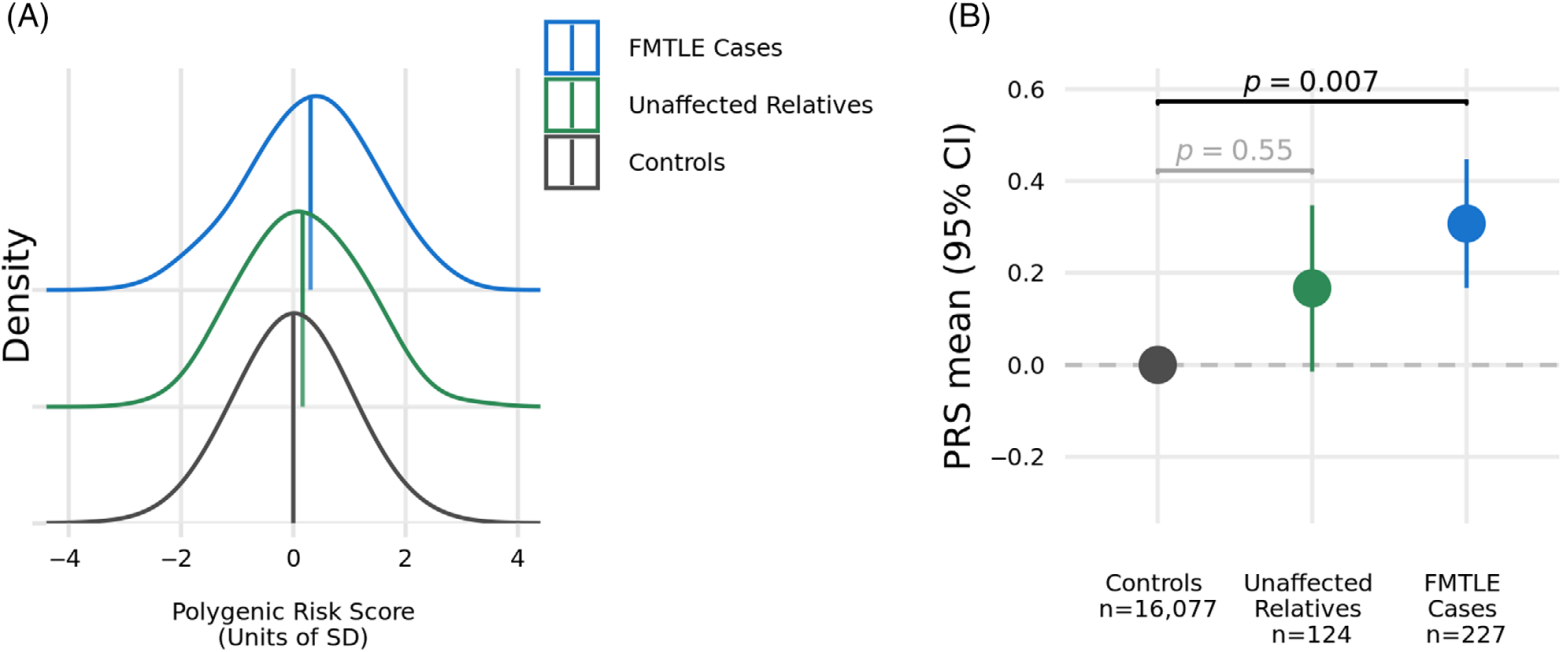
Comparison of mean PRS for focal epilepsy across affected individuals from FMTLE families, their unaffected relatives, and population controls. (A) Normalized distributions of focal epilepsy PRS. (B) Shown are the means of the normalized PRS values with 95% CIs for the FMTLE cases, their relatives, and population controls. CI = confidence interval; FMTLE = familial mesial temporal lobe epilepsy; PRS = polygenic risk score. [Color figure can be viewed at www.annalsofneurology.org]

Focal epilepsy polygenic risk was significantly higher in FMTLE cases compared to controls (odds ratio [OR] = 1.24, 95% confidence interval [CI] = 1.06, 1.46, *p* = 0.007; Fig. 2). Although the mean PRS for unaffected relatives was also elevated compared to controls, the difference was not significant (*p* = 0.55). No difference between FMTLE cases and their unaffected relatives was observed (*p* = 0.16).

**Figure 3:**
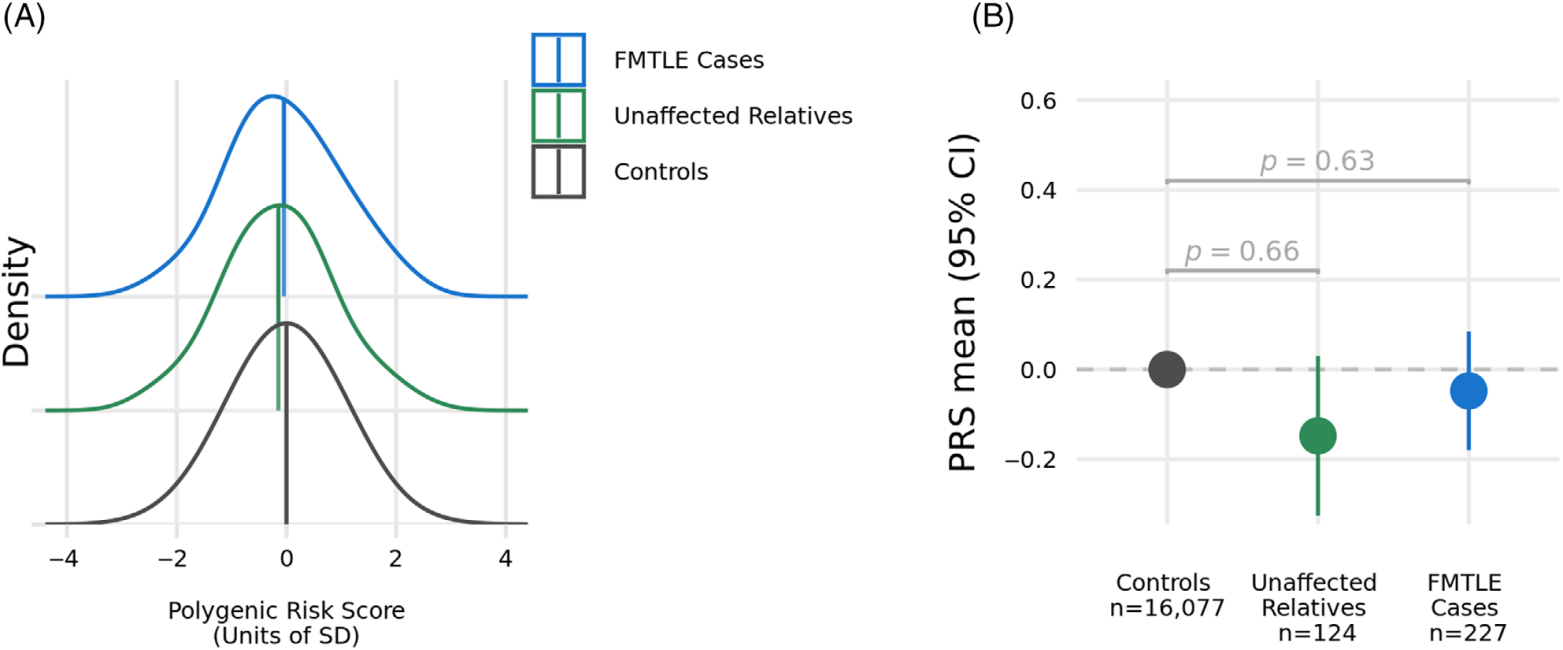
Comparison of mean PRS for FS across affected individuals from FMTLE families, their unaffected relatives and population controls. (A) Normalized distributions of FS‐PRS, showing standard deviation (SD). (B) Shown are the means of the normalized PRS values with 95% CIs for the affected cases from FMTLE families, their unaffected relatives, and population controls. CI = confidence interval; FMTLE = familial mesial temporal lobe epilepsy; FS = febrile seizure; PRS = polygenic risk score. [Color figure can be viewed at www.annalsofneurology.org]

No significant difference in FS polygenic risk was observed between FMTLE cases or unaffected relatives compared to population controls (OR = 1.04, 95% CI = 0.89, 1.22, *p* = 0.63) or for unaffected relatives compared to population controls (*p* = 0.66; Fig. 3). GGE polygenic risk trended lower for the FMTLE cases compared to population controls, however, the difference was not significant (OR = 0.86, 95% CI = 0.74, 1.01, *p* = 0.06).

## Discussion

We analyzed the contribution of common risk variants for focal epilepsy to our FMTLE cohort using PRS. We found that affected cases from FMTLE families had a higher mean PRS for focal epilepsy compared to population controls. There was a non‐significant trend for an elevated PRS in first or second‐degree unaffected relatives of FMTLE cases (see Fig. 2). Our findings provide the first genetic evidence that common variants contribute to the complex genetic basis of FMTLE, validating earlier clinical observations.^7^

Our recent study on PRS in families with all types of epilepsy demonstrated a role for common epilepsy risk variants, but when stratified for focal versus generalized epilepsy type, such a role appeared limited to familial genetic generalized epilepsies.^22^ In that study, within heterogeneous focal syndromes, we did not demonstrate an elevated focal epilepsy polygenic risk burden in cases with a family history of epilepsy (n = 601), nor in those with sporadic focal epilepsy (n = 675), compared to population controls.^22^

In contrast, here, we see significant enrichment of focal epilepsy polygenic risk in a cohort of FMTLE, despite the lack of genome‐wide significant SNPs in the focal epilepsy GWAS.^19^ This observation suggests that there are important risk variants for FMTLE that have not reached genomewide significance in the broader group included in the focal epilepsy GWAS possibly due to the heterogeneous nature of this group. Many focal epilepsies are acquired, some are due to rare germline variants or brain somatic mosaicism, and several familial focal epilepsy syndromes have been identified, each with a range of clinical phenotypes, inheritance patterns, and genetic heterogeneity.^20^ The heterogeneous nature of the focal epilepsies possibly limited the ability for any common variant enrichment to be detected in previous large GWAS and the familial epilepsy PRS study.^22^ Studying more homogeneous familial focal epilepsy cases, such as FMTLE, may be the route to identify specific genetic risk alleles.

In assessing the phenotypic homogeneity of our cohort, the clinical features in our large FMTLE cohort mirror those described in the recent ILAE classification of epilepsy syndromes^2^ drawing from our earlier studies^5^, ^7^ and those of others.^13^, ^14^ We confirm that an adolescent/young adult onset is usual, and that déjà vu is the most common focal seizure symptom. Other common symptoms include an epigastric rising sensation, fear/anxiety, nausea, and a dreamlike sensation. The majority of cases had a benign clinical course, with only 12% being pharmaco‐resistant.

Variations from this typical pattern can be observed in our data too. Familial cases with more severe MTLE, some with HS, drug‐resistance, and an earlier age of onset are known.^13^, ^14^ In total, we found 27 cases of MTLE with HS in our cohort (13% of those with available MRI results). An excess of cases with drug‐resistant MTLE and HS were observed in our unpublished families compared to our previously published ones,^5^, ^7^, ^8^, ^16^, ^33^ which we hypothesize is due to an increase in referrals for drug‐resistant patients. Many of our earlier families were ascertained via a combination of twin studies, routine clinical appointments, referral based on a positive family history of epilepsy, and deep phenotyping of all available family members, including those without a known history of epilepsy.^7^, ^8^ Our newer families were more frequently ascertained through tertiary hospitals which are more likely to receive referrals for patients with drug‐resistant epilepsy. It is unclear if these more severe cases are part of one phenotypic spectrum with the more common, milder cases, or if they represent a distinct syndrome. The fact that 5 of our families presented with several members with HS suggests there could be specific genetic determinants for this group, as suggested by Kobayashi et al.^13^

The average age of seizure onset varies between different studies of FMTLE.^7^, ^14^, ^34^ We found the mean age of afebrile seizure onset to be 21.6 years (range = 2.5–70 years, median = 18 years, IQR = 13–28 years), congruent with our previous studies.^7^, ^16^ Some hospital‐based studies of FMTLE found average ages of onset in early adolescence (median = 12 years, range = 3 months to 35 years^13^, ^14^). A recent report of 25 Mexican families with FMTLE reported a younger age of onset with a mean of 7 years.^34^ Age of seizure onset is a known familial epilepsy trait, regardless of epilepsy syndrome.^35^ Our cohort only included patients with European ancestry, and thus there may be ancestry‐specific genetic risk variants influencing age of onset in Mexican families that differ from our families.

Antecedent FS were reported in 16% of our FMTLE cases, compared to the estimated 2 to 5% of children that have FS under 5 years of age in European populations.^36^, ^37^ Furthermore, 52 individuals within our FMTLE families had FS alone, without later development of epilepsy, a pattern which has been reported previously.^15^, ^18^ Despite this, we found no enrichment of FS polygenic risk in our cases with MTLE. This is interesting given that a significant genetic correlation between FS and focal epilepsy common variants has been reported, supporting a shared polygenic basis.^19^ Therefore, our results suggest that the risk variants predisposing to FS alone are distinct from the common focal epilepsy risk variants implicated in FMTLE. The etiology of FS is incompletely understood, and there are multiple suggested mechanisms.^38^, ^39^, ^40^ Genetic studies have implicated fever susceptibility genes, neuronal excitability genes, and immune system genes in FS.^27^, ^41^, ^42^ Given we did not see shared common variants in FMTLE with FS, the presence of FS in FMTLE may represent an increased seizure susceptibility more broadly. Consistent with focal and generalized epilepsies following different genetic architecture,^19^, ^21^ our FMTLE cohort was not enriched for GGE PRS.

This study had limitations. It was not an epidemiologically designed study, and we cannot make conclusions about the incidence of FMTLE; previous data indicate that FMTLE is a common syndrome, accounting for almost 20% of new diagnoses of non‐lesional MTLE.^8^ Furthermore, only those of European descent were included in our genetic analyses, as the GWAS model used to generate PRS was completed using European samples only. PRS models do not generalize well across different ethnicities and larger, more diverse GWAS studies are required to address this disparity.^23^ More specific to this study, however, we note that our cohort, although broadly European, comprised of controls and families predominantly from Australia but also Italy. In doing so, this increased our cohort size and in turn study power. However, we cannot rule out the unintentional introduction of some population stratification with the Italian families despite controlling for genetic ancestry in our statistical tests. Finally, we note that the PRS applied here were an aggregate sum of 1,000 of mostly sub‐threshold GWAS SNPs to which the biological drivers remain unknown. Therefore, whereas the observation of focal epilepsy PRS enrichment for FMTLE provides general support for common risk variants playing a role, we do not know which specific variants, nor the genes they implicate.

In conclusion, patients with FMTLE largely have a mild, self‐limited, pharmaco‐responsive syndrome with prominent ictal déjà vu and adolescent/young adult seizure onset. The clinical spectrum of FMTLE includes rare cases of drug‐resistance, earlier age of onset, and HS, and these cases have been observed in small numbers in our study and earlier studies. Genetic background likely accounts for some of the clinical variation in FMTLE. Our findings provide support for a common polygenic basis of FMTLE. Our PRS analysis suggests that genetic determinants for FMTLE may be present in the sub‐threshold focal epilepsy GWAS SNPs and a future GWAS focused on this specific clinical group may well reveal these underlying loci.

## Author Contributions

R.V.H., K.L.O., P.P., S.A.M., I.E.S., M.B., and S.F.B. contributed to the conception and design of the study. R.V.H., K.L.O., P.P., P.S., A.L., A.R., B.E.G., J.R., J.H., M.T., T.J.O., P.K., L.G.S., E.M., D.E.C., I.E.S., C.N., A.G., and S.F.B. contributed to the acquisition and analysis of data. R.V.H, K.L.O., P.P., B.E.G., and S.F.B. contributed to drafting a significant portion of the manuscript or figures.

## Potential Conflicts of Interest

Nothing to report.

## Data Availability

Anonymized clinical data not published within this article will be made available by reasonable requests from any qualified investigator. Individual‐level SNP genotyping data is not available due to ethical restrictions; participants were not consented for genetic data sharing.
